# The Heterodimeric Glycoprotein Hormone, GPA2/GPB5, Regulates Ion Transport across the Hindgut of the Adult Mosquito, *Aedes aegypti*

**DOI:** 10.1371/journal.pone.0086386

**Published:** 2014-01-20

**Authors:** Jean-Paul Paluzzi, Mark Vanderveken, Michael J. O’Donnell

**Affiliations:** 1 Department of Biology, York University, Toronto, Ontario, Canada; 2 Department of Biology, McMaster University, Hamilton, Ontario, Canada; Rosalind Franklin University, United States of America

## Abstract

A family of evolutionarily old hormones is the glycoprotein cysteine knot-forming heterodimers consisting of alpha- (GPA) and beta-subunits (GPB), which assemble by noncovalent bonds. In mammals, a common glycoprotein hormone alpha-subunit (GPA1) pairs with unique beta-subunits that establish receptor specificity, forming thyroid stimulating hormone (GPA1/TSHβ) and the gonadotropins luteinizing hormone (GPA1/LHβ), follicle stimulating hormone (GPA1/FSHβ), choriogonadotropin (GPA1/CGβ). A novel glycoprotein heterodimer was identified in vertebrates by genome analysis, called thyrostimulin, composed of two novel subunits, GPA2 and GPB5, and homologs occur in arthropods, nematodes and cnidarians, implying that this neurohormone system existed prior to the emergence of bilateral metazoans. In order to discern possible physiological roles of this hormonal signaling system in mosquitoes, we have isolated the glycoprotein hormone genes producing the alpha- and beta-subunits (AedaeGPA2 and AedaeGPB5) and assessed their temporal expression profiles in the yellow and dengue-fever vector, *Aedes aegypti*. We have also isolated a putative receptor for this novel mosquito hormone, AedaeLGR1, which contains features conserved with other glycoprotein leucine-rich repeating containing G protein-coupled receptors. AedaeLGR1 is expressed in tissues of the alimentary canal such as the midgut, Malpighian tubules and hindgut, suggesting that this novel mosquito glycoprotein hormone may regulate ionic and osmotic balance. Focusing on the hindgut in adult stage *A. aegypti*, where AedaeLGR1 was highly enriched, we utilized the Scanning Ion-selective Electrode Technique (SIET) to determine if AedaeGPA2/GPB5 modulated cation transport across this epithelial tissue. Our results suggest that AedaeGPA2/GPB5 does indeed participate in ionic and osmotic balance, since it appears to inhibit natriuresis and promote kaliuresis. Taken together, our findings imply this hormone may play an important role in ionic balance when levels of Na^+^ are limited and levels of K^+^ are in excess – such as during the digestion and assimilation of erythrocytes following vertebrate blood-feeding by females.

## Introduction

In mammals, classic heterodimeric glycoprotein hormones include the pituitary and placental gonadotropins luteinizing hormone (LH), follicle-stimulating hormone (FSH) and choriogonadotropin (CG) along with thyroid-stimulating hormone (TSH) [1]. These hormones share a common alpha subunit, glycoprotein A (GPA1), and each contains a unique beta subunit, glycoprotein B (GPB1-4), which confers receptor specificity. A little over a decade ago, genome screening revealed the existence of another heterodimeric glycoprotein hormone, thyrostimulin, which consists of novel alpha (GPA2) and beta (GPB5) subunits [2]. As its name implies, thyrostimulin activates the TSH receptor, but does not act upon gonadotropin receptors [3].

In insects, heterodimeric glycoprotein hormones include buriscon, which plays a key role in melanization and cuticle hardening during development [4], [5]. With the recent discovery of two novel subunits forming an additional heterodimeric glycoprotein hormone in mammals, similar glycoprotein hormone forming subunits have been identified in insects [2], [6]. Recently, an elegant study using the fruit fly genetic toolbox revealed that the glycoprotein hormone subunits are most highly expressed in a bilateral pair of neuroendocrine cells within each of the first four abdominal neuromeres in both larvae and adults [7].

Glycoprotein hormone receptors belong to the large G protein coupled receptor (GPCR) superfamily, but are unique in that they contain a large extracellular amino-terminus with multiple leucine-rich containing repeats. This large ectodomain is essential for facilitating interaction with their natural glycoprotein hormone ligands [8]–[11]. Two glycoprotein hormone receptors have been identified in the fruit fly, *Drosophila melanogaster*. The first to be identified, *Drosophila* leucine-rich repeat containing GPCR (DLGR1), has highly similar structural homology to the mammalian glycoprotein hormone receptors [12], although its natural ligand had not yet been elucidated at the time. It was later shown that the fly heterodimeric glycoprotein hormone GPA2/GPB5 stimulates DLGR1 and elevates cAMP levels in transfected HEK293T cells [6]. DLGR1 displays expression soon after oviposition that continues throughout all developmental stages with enrichment in adult male flies [12]. The second fruit fly glycoprotein hormone receptor, DLGR2, was subsequently identified and proved to be the receptor for bursicon [4], [5]. Unlike bursicon, whose biological role has been known for over half a century [13], [14], the role of the GPA2/GPB5 hormone in insects remains unclear. In the fruit fly, DLGR1 is enriched in epithelial tissues such as the hindgut, salivary glands and Malpighian tubules [15] and shows sex-specific variation with six-fold higher expression in adult males relative to females [12]. Taken together with the observation that GPA2/GPB5 neuroendocrine cells are large and project processes from the segmental nerves to the abdomen of the fly, this led to the suggestion that this ancient neurohormone system may modulate ion and osmotic balance in insects [7]. A similar role may also occur in more distantly related invertebrates since GPA2 is known to regulate intestinal activity in the worm, *Caenorhabditis elegans* [16].

With an interest in elucidating novel neuroendocrine systems regulating physiological activities in blood-feeding arthropods, we set out to isolate and characterize the GPA2/GPB5 homologous neuroendocrine signaling system in the mosquito, *Aedes aegypti*. Using conditioned medium from cultured cells expressing the mosquito recombinant glycoprotein hormone, we have identified a novel physiological target, which also exhibits enriched expression of the putative AedaeGPA2/GPB5 glycoprotein receptor, AedaeLGR1. Moreover, the glycoprotein hormone subunits and the glycoprotein hormone receptor demonstrate expression profiles that are elevated during the terrestrial-dwelling adult stage. Taken together, these finding reveal that this evolutionarily old neurohormonal system may play an *in vivo* role related to the maintenance of ionic and osmotic balance in this principal vector of dengue and yellow-fever viruses.

## Methods

### Animals

A colony of *Aedes aegypti* (Liverpool) was established at McMaster University in the Department of Biology using eggs obtained from Dr. Andrew Donini (Biology, York University). Eggs were hatched in plastic storage containers filled with distilled water. Larvae were fed daily with several drops of a 2% solution of liver powder and yeast (1:1) and larval baths were maintained in an incubator at 28°C on a 12:12 hour light:dark cycle. Pupae were transferred into small plastic petri dishes within hardwood-framed adult cages lined with mosquito netting. Adults were provided with a 10% sucrose solution and females were regularly fed sheep blood in Alsever’s solution (Cedarlane Laboratories Ltd., Hornby, ON) for colony maintenance.

### Cloning of Glycoprotein Hormone Subunits and the Leucine-rich Repeating-containing GPCR (LGR1)

The *A. aegypti* glycoprotein hormone subunits and receptor homologs were sought within the nucleotide databases on VectorBase (www.vectorbase.org) using the *D. melanogaster* glycoprotein GPA2 (NP_001104054), GPB5 (EDP28157) and DLGR1 receptor (AAB07030) sequences as queries. Hypothetical transcripts or EST hits for *A. aegypti* GPA2 (GenBank accession: BN001241), GPB5 (GenBank accession: XM_001653331), and LGR1 (GenBank accession: XM_001648982) were documented and primers designed encompassing the entire open reading frame (see Table 1). Preparation of cDNA template for PCR involved RNA isolated using the Purelink RNA mini kit (Ambion, Austin, TX) from whole adult *A. aegypti* <24 hours following eclosion to an adult. Preparation of cDNA used for amplification of *A. aegypti* GPA2 and GPB5 was synthesized using an oligo dT primer and following manufacturer recommendations in the iScript Select Reverse Transcriptase system (Bio-Rad, Mississauga, ON). Preparation of cDNA for amplification of *A. aegypti* LGR1 involved a gene-specific primer (see Table 1) and followed recommendations for cDNA synthesis using the iScript Select Reverse Transcriptase system (Bio-Rad, Mississauga, ON). Amplicons were obtained following manufacturer cycling conditions using a high-fidelity Platinum Taq DNA polymerase (Life Technologies, Burlington, ON). Glycoprotein hormone subunits and LGR1 cDNA were cloned into pGEM T-Easy vector (Promega, Madison,WI) and sequences from several independent amplicons were sequenced to ensure base accuracy at the MOBIX Lab Sequencing Facility (McMaster University, Hamilton, ON). The consensus *A. aegypti* LGR1 cDNA was compared against the genomic scaffold sequences that were imported into Geneious Software 6.1.6 to prepare an organizational map of the receptor gene.

**Table 1:** Primers designed for initial cloning of cDNA of glycoprotein hormone subunits and its leucine-rich repeat-containing GPCR.

Oligonucleotide name:	Oligonucleotide sequence:	Target and usage:
Aedes GPA2 F1	ATGGAGTGGCTACGATTTGC	sense primer for amplification of A. aegypti GPA2 predicted cDNA
Aedes GPA2 R1	TCAGTCCTTTTTACAGTGGTAACAC	anti-sense primer for amplification of A. aegypti GPA2 predicted cDNA
Aedes GPA2 kozak	GCCACCATGGAGTGGCTACGATTTGC	sense primer introducing Kozak sequence into A. aegypti GPA2 predicted cDNA
Aedes GPA2 HIS-tag	TCAATGGTGATGGTGATGATGGTCCTTTTTACAGTGGT	anti-sense primer for incorporation of hexa histidine-tag on carboxyl terminus of GPA2
Aedes GPB5 F1	ATGATCAATCTAATCTCGGTATGGA	sense primer for amplification of A. aegypti GPB5 predicted cDNA
Aedes GPB5 R1	CTAAGCGTATTCGGATTCGATTT	anti-sense primer for amplification of A. aegypti GPB5 predicted cDNA
Aedes GPB5 kozak	GCCACCATGATCAATCTAATCTCGGT	sense primer introducing Kozak sequence into A. aegypti GPB5 predicted cDNA
Aedes GPB5 HIS-tag	TCAATGGTGATGGTGATGATGAGCGTATTCGGATTCGA	anti-sense primer for incorporation of hexa histidine-tag on carboxyl terminus of GPB5
Aedes LGR1 F1	ATGAGCGTATGTAGTGCACGG	sense primer for amplification of A. aegypti LGR1 predicted cDNA
Aedes LGR1 R1	TTACAGAAAAGTTTCACTTTCCTGC	anti-sense primer for cDNA synthesis andamplification of A. aegypti LGR1 predicted DNA

### Mosquito GPA2, GPB5 and LGR1 Deduced Protein Sequence Analysis

The *A. aegypti* GPA2 and GPB5 deduced protein sequence was assessed for secretory protein potential by examining for the presence of a signal peptide using SignalP4.0 [17]. AedaeGPA2 has a predicted signal peptide with cleavage predicted between Ala_18_ and Arg_19_ residues. Similarly, AedaeGPB5 also has a predicted signal peptide and its cleavage site is predicted between Ala_18_ and Val_19_ residues. Detection of putative N-linked glycosylation sites was carried out using NetNGlyc 1.0 (http://www.cbs.dtu.dk/services/NetNGlyc) [18].

The *A. aegypti* LGR1 deduced protein sequence was analyzed for predicted membrane topology using the GPCRHMM transmembrane prediction server [19] and detection of N-linked glycosylation sites predicted as noted above. AedaeLGR1 was compared to related receptor proteins from other insects and structurally related glycoprotein hormone receptors from mammals using ClustalW analysis in MEGA 5.2 [20]. The evolutionary relationships among the selected insect glycoprotein hormone and classic mammalian glycoprotein hormone receptors was inferred using neighbor-joining method [21] and maximum likelihood method based on the JTT matrix-based model [22], which yielded trees with identical topologies. The reliability of the relationships between the taxa was tested using bootstrap analysis with 1,000 iterations [23].

Structural homology modeling analysis for the AedaeLGR1 was carried out using the SwissModel server (www.swissmodel.expasy.org) running automatic modeling mode [24]–[26]. Two regions of AedaeLGR1 that were structurally modeled in this analysis include the large ectodomain and transmembrane spanning hydrophobic domains spanning residues Lys_95_ to Ala_363_ and Leu_558_ to Leu_838_, respectively. Under the automatic modeling mode, the templates that were selected for modeling the major structural features of AedaeLGR1 were the human FSH ectodomain (Protein Data Base accession code: 4ay9) that includes the leucine-rich repeat-containing region and hinge region, the latter of which was recently shown not to form a distinct structural entity [9]. The transmembrane spanning domains of AedaeLGR1 were modeled based on the squid rhodopsin structure (Protein Data Base accession code: 2ziy) [27].

### Preparation of Expression Constructs for GPA2/GPB5 Hormone Expression

In order to facilitate detection of the two subunits in CHO-K1 cells, each subunit cDNA was modified to include a hexa histidine-tag on the carboxyl terminus (see Table 1). In addition, a Kozak initiation sequence was introduced into each expression clone as described previously [28] to optimize expression in the mammalian CHO-K1 cells [29]. For expression of single subunits, clones were inserted into pcDNA3.1; for dual expression of both subunits, clones were introduced into the dual promoter plasmid, pBud-CE4.1 obtained from Addgene (plasmid#23027; [30]).

### Transient Expression and Purification of Mosquito GPA2/GPB5 Hormone

CHO-K1 cells were maintained as previously described [31] and were grown in T75 culture flasks (Corning, Tewksbury, MA) in a water-jacketed incubator at 37°C, 5% CO_2_. Cells were transfected at 80–90% confluency in basal media containing only antibiotic-antimycotic with FuGene HD transfection reagent (Promega, Madison, WI) using a 2:1 reagent to plasmid DNA (µg) ratio. Conditioned media from transfected cells was collected 48–72 hours later and was concentrated using a 3 kDa MWCO Macrosep Advance Centrifugal Device (Pall, Ann Arbor, MI). The heterodimeric hormone and/or individual subunits were subsequently purified using the MagneHIS Protein Purification System (Promega, Madison, WI) following recommended guidelines for purification of secreted proteins. The high imidazole concentration in the elution buffer was reduced by diluting 50-fold in phosphate buffered saline containing 0.02% sodium azide. This larger volume containing the purified heterodimeric hormone or individual subunits was concentrated using a centrifugal concentrating device as noted above.

### Protein Quantification and Western Blot Analysis

The final purified protein concentration was determined by a micro scale Bradford assay [32] using bovine serum albumin standard (Bio-Rad Laboratories, Mississauga, ON) and measured with a Nanodrop 1000 spectrophotometer (Thermo Fisher Scientific, Wilmington, DE). Samples of purified recombinant protein along with protein standards were resolved using 15% SDS–polyacrylamide gel electrophoresis under reducing conditions. Following electrophoresis, samples were transferred to polyvinylidene difluoride (PVDF) membrane (Millipore, Bedford, MA) using a wet transfer system (Bio-Rad Laboratories, Mississauga, ON). Following transfer, membranes were rinsed for 5 minutes at room temperature (RT) in phosphate buffered saline containing 0.1% Tween-20 (PBST). The PVDF membranes were blocked with PBST containing 3% skim milk powder (PBSTB) for 1 hour at RT. Membranes were then incubated overnight at 4°C in PBSTB containing anti-HIS tag mouse monoclonal antibody (Applied Biological Materials, Richmond, BC) diluted 1:2000. The next day, membranes were washed in fresh PBST at RT for a total of 1 hour with fresh PBST replaced every 20 minutes. Membranes were then incubated in PBSTB containing anti-mouse HRP conjugated secondary antibody (Promega, Madison, WI) for 1 hour at RT at a 1:6000 dilution. Finally, membranes were washed for 1 hour at RT with PBST buffer exchange every 20 minutes. For detection, blots were drained of excess PBST and incubated with Clarity Western ECL (Bio-Rad, Mississauga, ON) substrate for 5 minutes. Images were acquired using a ChemiDoc MP Imaging System following recommended settings (Bio-Rad, Mississauga, ON).

To confirm whether the *A. aegypti* glycoprotein subunits are glycosylated, purified recombinant protein (∼10 ug) was diluted with DTT buffer and denatured by incubating at 55°C for 10 minutes. Once denatured, the recombinant proteins were added to a deglycosylation reaction containing G7 Reaction buffer and 225 units of Remove-iT PNGase F (New England Biolabs, Whitby, ON) and incubated at 37°C for 1 hour. Control reactions were treated similarly, but did not include PNGase-F enzyme.

### Spatial and Temporal Expression Analysis by Reverse-transcriptase Quantitative PCR

All post embryonic stages were isolated and used for RNA extraction using the PureLink RNA Micro Kit (Life Technologies, Burlington, ON) following the manufacturer’s guidelines and purified RNA was quantified on a Nanodrop 1000 spectrophotometer (Thermo Fisher Scientific, Wilmington, DE). Prior to cDNA synthesis, 1 µg RNA was treated with DNase I, RNase-free (Fisher Scientific, Ottawa, ON) following the suggested protocol to remove any potential genomic DNA present in the RNA samples. Following DNase treatment, 0.5 µg RNA from each developmental stage was used for cDNA synthesis using the iScript cDNA Select synthesis kit and supplied Oligo dT primer (Bio-Rad, Mississauga, ON). For tissue expression analysis in adult stage *A. aegypti*, insects were dissected in chilled RNase-free PBS and tissues were processed for RNA isolation and subsequent cDNA synthesis as noted above.

Primers for qPCR were designed over exon-exon boundaries whenever possible to ensure amplification of cDNA only. Five candidate reference genes were examined for optimal expression stability in both temporal and adult spatial expression analyses using Bestkeeper [33] and Normfinder [34] plugins for Microsoft Excel as described previously [31]. Reference gene primers were based on gene predictions and included beta-actin (GenBank accession: XM_001655125), alpha-tubulin (GenBank accession: XM_001663357), ribosomal protein 49 (GenBank accession: AY539746), 40S ribosomal protein L8 (GenBank accession: XM_001657661), and 60S ribosomal protein S18 (GenBank accession: XM_001660270). Primer information for all qPCR targets are provided in Table 2; however, data were normalized to the geometric average [35] of the three most stable reference genes, namely beta-actin, 40S ribosomal protein L8 and 60S ribosomal protein S18. Experiments were repeated with two biological replicates each having duplicate technical replicates to ensure reproducibility of relative expression results.

**Table 2:** Primers designed for quantitative PCR analysis of several candidate target genes for determining suitability as reference genes as well as the GPA2/GPB5 glycoprotein hormone subunits and its cognate LGR1 receptor in *Aedes aegypti*.

Oligonucleotide name:	Oligonucleotide sequence:	Product size (bp):	GenBank accession number:
Actin-F	ACCGATATCAACATGTGTGACG	211	XM_001655125
Actin-R	TCAAGATACCTCGTTTGCTCTG		
Alpha-tubulin-F	ACTTCTGCTTCAAAATGCGTG	237	XM_001663357
Alpha-tubulin-R	CTACGGTTGGTTCCAGATCG		
rp49-F	ACAAGCTTGCCCCCAAC	214	AY539746
rp49-R	GCGATTTCGGCACAGTAGA		
rpL8-F	AACCGTCAAGCAAATCATCC	238	XM_001657661
rpL8-R	GTCACCGGTCTTCTCCTCC		
rpS18-F	TAAAAATGTCGCTCGTGATCC	249	XM_001660270
rpS18-R	AATCGGGGATCTTGTACTGG		
LGR1-F	GCCGGTTGCGTATCTTTTC	290	KF711859
LGR1-R	ATCAAATGTTGTGGGCGTAAG		
GPA2-F	CCACAAAGTAGGGCACACG	267	BN001241
GPA2-R	TGGTAACACGAGCAGTTGG		
GPB5-F	CGTGAGCGTGTGGTCTTG	234	BN001259
GPB5-R	CCCTCACAGCTAGTATCCATGC		

### Dissection and Preparation of Hindgut Tissue for Monitoring Ion Transport

Adult mosquitoes of both sexes were dissected under Ca^2+^-free *Aedes* saline consisting of 153.4 mM NaCl, 3.4 mM KCl, 1.8 mM NaHCO_3_, 1 mM MgSO_4_, 25 mM HEPES, and 5 mM glucose adjusted to pH 7.1. Forceps were used to grip the last abdominal segment, tear open the cuticle, and gently pull the hindgut free from the body cavity. The dissected hindgut was transferred from the dissection dish to a 35 mm Petri dish (BD Biosciences Canada, Mississauga, ON) containing *Aedes* saline held within a hot melt glue enclosure to facilitate the use of small saline volumes. The 35 mm Petri dishes were pre-coated with 70 µl of 62.5 µg ml^−1^ poly-L-lysine (70–150 kDa, Sigma-Aldrich, Oakville, ON) and air dried before filling with saline. This treatment faciliatated adherence of the dissected tissue to the bottom of the dish.

### Ion-selective Microelectrodes

Micropipettes were pulled from 1.5 mm borosilicate glass capillaries (World Precision Instruments Inc., Sarasota, FL) using a P-97 Flaming/Brown pipette puller (Sutter Instruments Co., Novato, CA). The resulting micropipettes had a tip opening of ∼5 µm and were characterized by a short shank. Micropipettes were silanized with 50 µl of dichlorodimethylsilane applied to the interior of a glass Petri dish which was inverted over a group of approximately 20–30 micropipettes on the surface of a hot plate at 200°C. Silanized micropipettes were transferred to a desiccator after 30 minutes and stored until use.

Silanized micropipettes were made selective for K^+^ ions by backfilling them with 150 mM KCl then tip-filling with a column (∼500 µm) of K^+^ ionophore I, cocktail B (Fluka, Buchs, Switzerland). Similarly, silanized micropipettes were made selective for Na^+^ ions by backfilling with 150 mM NaCl then tip-filling with a column (∼200 µm) of Na^+^ ionophore cocktail consisting of 3.5% Na^+^ ionophore X (Fluka, Buchs, Switzerland), 95.9% 2-nitrophenyl octyl ether, and 0.6% potassium tetrakis (4-chlorophenyl) borate. Micropipettes were backfilled using a plastic 1 ml syringe pulled out to a fine tip over a low flame [36]. The K^+^ ionophore cocktail has greater selectivity for K^+^ than for Na^+^, Ca^2+^, or Mg^2+^ by factors of 10^3.9^, 10^4.9^, and 10^4.9^, respectively [37]. A similar Na^+^ ionophore cocktail (comprised of 10% Na^+^ ionophore X, 89.75% nitrophenyl octyl ether, and 0.25% sodium tetraphenylborate) to that used in the present study has greater selectivity for Na^+^ than for K^+^, Ca^2+^, or Mg^2+^ by factors of 10^2.6^, 10^3.5^, and 10^3.7^, respectively [38]. Later it was show that the lower ratio between the lipophilic ion (potassium tetrakis (4-chlorophenyl) borate) and the ionophore in the cocktail used for the present study increased the stability of the microelectrode’s Nernst slope over time while maintaining favourable selectivity for Na^+^ over other cations [39]. Reference electrodes were produced from 8 cm borosilicate glass filamented capillaries filled with boiling 1 M KCl containing 3% agar. Reference electrodes were immersed in 1 M KCl at 4°C until use.

### Scanning Ion-selective Electrode Technique (SIET)

The ion-selective microelectrode was connected to an amplifier headstage whose motion was controlled by three computerized stepper motors, which moved the electrode along the X, Y, and Z-axes with submicron accuracy and repeatability. Automated Scanning Electrode Technique (ASET) software version 2.0 (Science Wares, Falmouth, MA) and hardware from Applicable Electronics (Forestdale, MA) were used to make SIET measurements and control the motion of the stepper motors. Ion-selective microelectrodes were calibrated prior to scanning each tissue. K^+^-selective microelectrodes were calibrated in solutions of 150 mM KCl and 15 mM KCl with 135 mM NaCl. Na^+^-selective microelectrodes were calibrated in solutions of 150 mM NaCl and 15 mM NaCl with 135 mM KCl. Slopes for a ten-fold change in ion concentration were (mean±SEM) 55.6±0.3 (N = 20) for K^+^-selective microelectrodes and 59.0±0.2 (N = 20) for Na^+^-selective microelectrodes.

Measurements of voltage gradients were made by moving the microelectrode tip perpendicularly to the tissue surface between two points separated by 50 µm. Each measurement consisted of a series of computer-controlled motions of the electrode. Initially, the microelectrode tip was positioned within 5 µm of the tissue surface, representing the inner limit of the microelectrode’s 50 µm excursion. Positioning was followed by a 4.0 second wait period during which no measurements were made to allow the re-establishment of ion gradients at the tissue surface following the localized disturbance due to the microelectrode movement. It was previously found that a wait time of at least 3 seconds was sufficient to allow the gradients to fully re-establish [40]. The voltage at the microelectrode tip was recorded for 0.5 seconds following the wait period. The microelectrode was then moved at 200 µm s^−1^ to a position at the outer limit of the 50 µm range where another wait and sample period was completed. The move, wait, and sample cycle encompassing recordings at both extremes of the microelectrode excursion was completed in ∼9.5 seconds and was repeated three times. Two independent measurements (each consisting of three move, wait, and sample cycles) were made consecutively at each tissue site, requiring a total of ∼57 seconds.

### SIET Measurements

Each scan consisted of measurements at sites in the posterior ileum and the rectum. Sites were separated by 40 µm, and the number of measurement sites varied between preparations based on the total length of the dissected tissue such that measurements were made over the majority of its length. In order to help reduce spontaneous contractions of the tissue, preparations were bathed in Ca^2+^-free *Aedes* saline during K^+^ measurements. Na^+^ measurements were also conducted with preparations bathed in a Ca^2+^-free *Aedes* saline but to improve the signal to noise ration, the 20 mM Na^+^ was made up by equimolar substitution with N-methyl-D-glucamine. A 10 µl volume of DPBS with 0.02% sodium azide (control) or recombinant mosquito GPA2/GPB5 in DPBS with 0.02% sodium azide were added to 190 µl of bathing saline ∼1 minute prior to SIET measurements. In the latter group, GPA2/GPB5 was added so that the final concentration was 200 nM. Following addition of saline alone or saline containing recombinant GPA2/GPB5, the microelectrode tip was positioned at a reference site located several hundred micrometers perpendicularly away from the tissue. At this site, which is sufficiently distant from the tissue that the influence of epithelial ion flux is negligible, the voltage gradient was recorded across the microelectrode’s 50 µm excursion in the same manner as when making measurements at the tissue surface. Although this voltage gradient should, in theory, be negligible, it is subtracted from the voltage gradients measured at the tissue surface in the calculation of epithelial ion flux to correct for microelectrode noise and any voltage drift during scanning. Measurements at the reference site commenced within 5 minutes of tissue dissection and required approximately 5 minutes to complete.

SIET measurements of the posterior ileum and rectum commenced after the completion of reference scans. For each preparation, either 4–5 sites were scanned on the posterior ileum, or 6–18 sites were scanned on the rectum. The rectum was divided into anterior and posterior regions by dividing the total number of measurement sites into anterior and posterior halves. Up to 32 minutes were required to complete SIET measurements on each tissue sample.

For K^+^ measurements, the average voltage gradient at the reference site was (mean±SEM) −13.1±4.6 µV (N = 10) for control samples and −1.0±3.9 (N = 10) for preparations treated with saline containing GPA2/GPB5. The average gradient at the tissue surface in each of these treatments was 36.3±7.8 µV (N = 10) and 67.7±14.6 µV (N = 10), respectively. The average reference site voltage gradient for Na^+^ measurements was −23.1±4.8 µV (N = 10) in controls and −23.6±6.7 µV (N = 10) in preparations treated with GPA2/GPB5. For Na^+^ measurements, the average gradient at the tissue surface was −45.1±16.2 µV (N = 10) for controls and −18.6±7.8 µV (N = 10) for the GPA2/GPB5 treatment. Non-zero voltage gradients at the reference site are usually attributable to slow drift of the voltage of either the ion-selective or reference microelectrodes caused by slow evaporation of the backfilling solution. The ratio between the voltage gradient recorded at the tissue surface and at the reference site suggests the degree of confidence with which the voltage signal can be attributed to transport of the ion of interest across the epithelium. Given the average voltage gradients at the tissue surface and at the reference site, the signal:noise ratio for K^+^-selective microelectrodes ranged from 3:1 to 6:1 in the posterior ileum and from 3:1 to 4:1 in the rectum. Similarly, the signal:noise ratio for Na^+^-selective microelectrodes was typically ∼2:1 in the posterior ileum and ranged from 0.3:1 to 0.7:1 in the rectum. Small peristaltic contractions of the preparation may have disturbed the unstirred layer during some SIET scans, leading to an underestimation of voltage gradients at the tissue surface by ion-selective microelectrodes.

### Calculation of Ion Flux

The voltages recorded at the ends of the 50 µm range were amplified 1000-fold and then the voltage difference between the two points was calculated. A standard microelectrode calibration curve relating voltage to ion activity was used to convert the voltage difference across the 50 µm excursion to K^+^ or Na^+^ activity. The equation [ΔC = C_B_10^(ΔV/S)^ −C_B_] was used to convert the voltage gradient into a concentration gradient for K^+^ or Na^+^. ΔC represents the concentration gradient in µmol cm^−3^; C_B_ represents the background ion concentration (the average of the concentrations at all points) in µmol cm^−3^; ΔV represents the voltage gradient measured at the tissue surface less the voltage gradient at the reference site in µV; S is the slope of the electrode in µV. Although ion-selective microelectrodes measure ion activity rather than concentration, data can be expressed in units of concentration if the activity coefficient for both the calibration and experimental solutions is assumed to be similar. This assumption is valid because the saline and calibration solutions are of similar ionic strength.

Fick’s first law of diffusion was used to calculate the K^+^ flux based on the concentration gradient through the equation [J_I_ = (D_I_ΔC)Δx^−1^]. J_I_ represents the net flux of the ion in pmol cm^−2^ s^−1^; D_I_ is the diffusion coefficient of the ion (1.92*10^−5^ cm^2^ s^−1^ for K^+^; 1.55*10^−5^ cm^2^ s^−1^ for Na^+^) [41]; ΔC represents the concentration gradient in µmol cm^−3^; Δx represents the excursion distance expressed in centimeters separating the points where voltage was recorded by the microelectrode.

### Graphing and Statistics

All data were compiled using Microsoft Excel and transferred into GraphPad Prism 4.0 for preparation of figures and statistical analysis. Data were analyzed using one-way ANOVA with Bonferroni’s multiple comparison post-test where differences between treatments were considered significant if *p*<0.05.

## Results

### Cloning of Mosquito Glycoprotein Hormone Subunit cDNA and Deduced Protein Sequence Analysis

**Figure 1:**
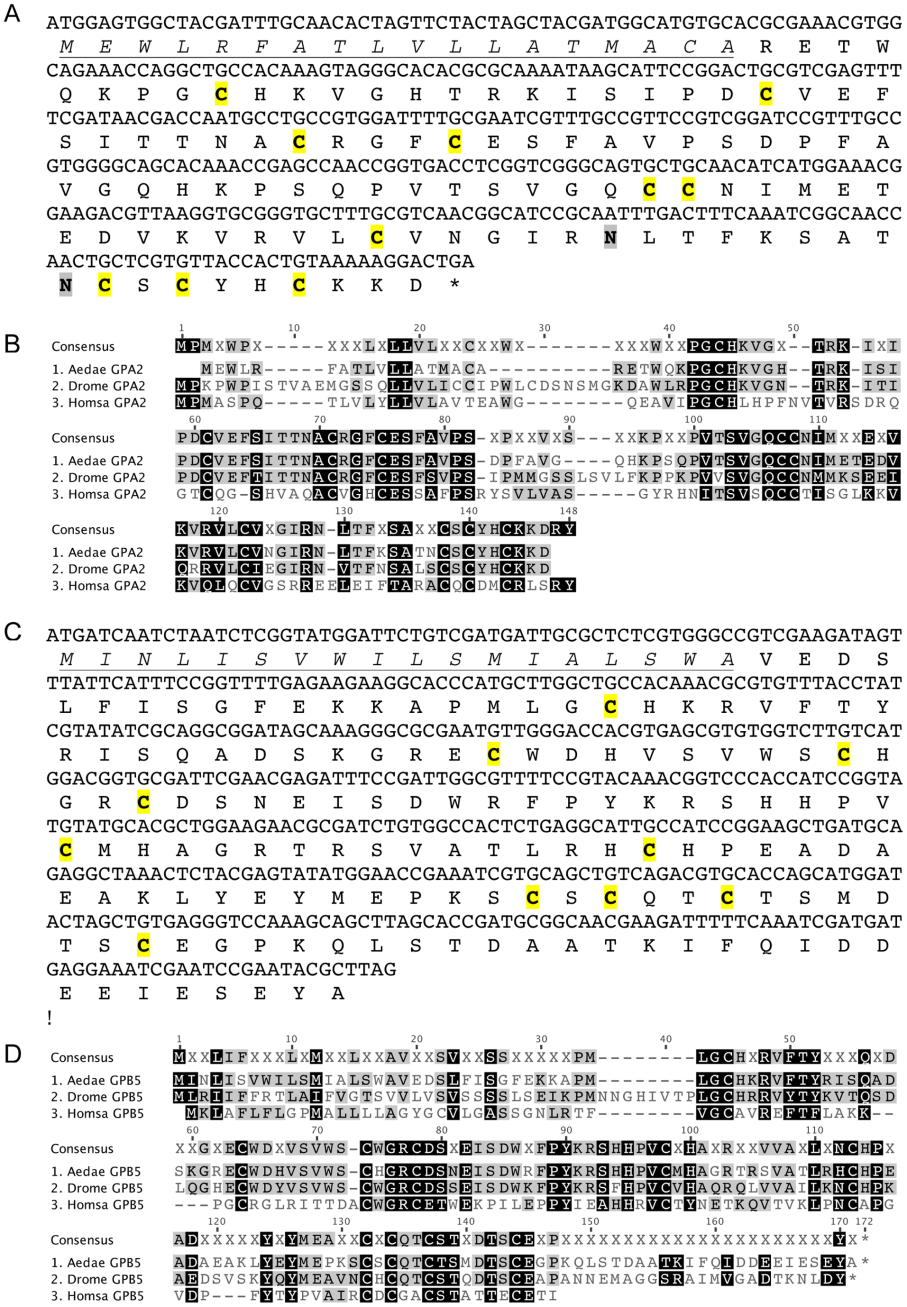
Mosquito glycoprotein hormone subunit cDNAs and deduced protein sequence. (A and C) *A. aegypti* GPA2 and GPB5 cDNAs and deduced amino acid sequences with the predicted signal peptide sequences underlined and italicized and putative N-linked glycosylation sites indicated in bold shaded font (no sites predicted in AedaeGPB5). Notably, 10 conserved cysteine residues in each subunit (A and C) forming disulfide bridges are denoted in bold highlighted font. Sequence alignment of mosquito GPA2 (B) and GPB5 (D) precursor proteins with their human and fruit fly counterparts demonstrating the conservation of key cysteine residues important for cystine-knot formation.

Utilizing the *D. melanogaster* glycoprotein subunits as queries, we searched the *A. aegypti* genome and predicted transcript database for the homologs of GPA2 and GPB5. We confirmed the cDNAs including a complete open reading frame (ORF) and the deduced amino acid sequence for each glycoprotein hormone subunit (Figure 1A). The *A. aegypti* GPA2 (AedaeGPA2) cDNA was compared to the genomic data and revealed that the AedaeGPA2 gene contains at least two exons (separated by a 14,378 bp intron) and spans over 14.7 kb of the genome (data not shown). This cDNA yields a deduced protein sequence of 120 amino acids with a highly predicted signal peptide with cleavage most likely occurring between Ala_18_ and Arg_19_ (Figure 1A). The mature AedaeGPA2, which has a predicted molecular weight of 11.3 kDa, contains 10 highly conserved cysteine residues that are required for forming a cystine-knot structure characteristic of glycoprotein hormones [42]. In addition, there are two predicted N-linked glycosylation sites that occur at residues Asn_103_ and Asn_111_ (Figure 1A). Primary structure comparison of the AedaeGPA2 sequence with the fruit fly and human homologs confirms the conservation of key cysteine residues that result in the formation of disulfide bridges. AedaeGPA2 shares 53.2% identity with the DromeGPA2 glycoprotein subunit while sharing 30.7% identity with human GPA2 glycoprotein subunit (Figure 1B).

The *A. aegypti* GPB5 (AedaeGPB5) cDNA was also compared to the genomic data to reveal that the AedaeGPB5 gene also contains at least two exons (separated by a 4392 bp intron) and spans at least 4.8 kb of the genome (Figure 1C), which is positioned on the opposite strand to the AedaeGPA2 gene with less than a 9.9 kb intergenic region. This second cDNA yields a deduced protein sequence of 162 amino acids with a highly predicted signal peptide with a most probable cleavage site occurring between Ala_18_ and Val_19_ (Figure 1C). The mature AedaeGPB5 has a predicted molecular weight of 16.5 kDa and also contains 10 highly conserved cysteine residues important for the formation of the cystine-knot structure characteristic of this family of proteins [42]. Unlike the AedaeGPA2 subunit, there are no predicted N-linked glycosylation sites occurring in the mature protein (Figure 1C). Comparison of the AedaeGPB5 deduced amino acid sequence with the fruit fly and human GPB5 counterparts confirms the conservation of key cysteine residues forming disulfide bridges that are involved in the characteristic cystine-knot structure. AedaeGPB5 shares 44.1% identity with the DromeGPB5 glycoprotein subunit and shares only 24.1% identity with human GPB5 glycoprotein subunit (Figure 1D).

### Recombinant Mosquito Glycoprotein Hormone Subunit Detection by Immunoblotting

**Figure 2:**
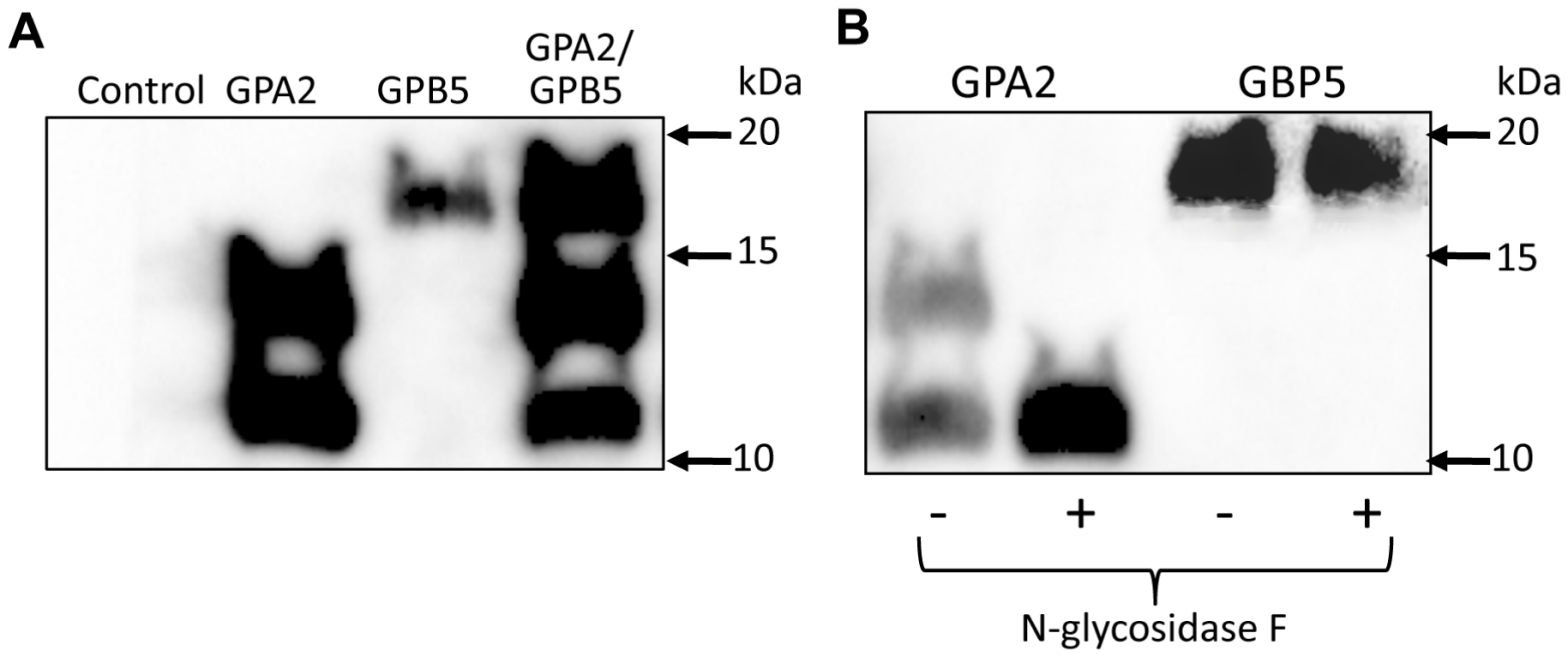
Western blot analysis of recombinant mosquito GPA2/GPB5. (A) Affinity-purified recombinant glycoprotein hormone was run in SDS-PAGE under reducing conditions and following immunoblotting, individual subunits were represented by two main bands in GPA2 samples (11.5 and 14.5 kDa), a single band in GPB5 samples (17.5 kDa) and three bands corresponding to the individual subunit bands when both subunits were coexpressed in CHO-K1 cells (see Methods). In (B), individual subunit samples were treated with PNGase F (N-glycosidase F) to determine if the subunit contained asparagine-linked carbohydrate side chains indicative of glycosylation during post-translational modification. Only the mosquito GPA2 subunit undergoes N-linked glycosylation as treatment with PNGase F eliminates the 14.5 kDa band and intensifies the band present at 11.5 kDa.

We expressed the subunits individually or together in CHO-K1 cells and determined that under reducing conditions, the two affinity purified subunit samples were detected by immunoblotting as two bands for AedaeGPA2 at approximately 11.5 and 14.5 kDa and only a single band for AedaeGPB5 at approximately 17.5 kDa (Figure 2A). No bands were detected in the affinity purified conditioned media sample from CHO-K1 cells transfected with empty vector. The smaller band for AedaeGPA2 approximates the molecular weight of the native protein including the addition of the polyhistidine tag on the carboxyl terminus. The higher molecular weight band detected by immunoblotting suggests this subunit may undergo post-translational modification, which may include the predicted N-linked glycosylation. For the AedaeGPB5 recombinant protein, the band migration is in line with predicted molecular weight including the polyhistidine tag on the carboxyl terminus. In order to confirm if the *A. aegypti* glycoprotein hormone subunits undergo sugar molecule attachment (i.e. glycosylation) during post-translational modification, affinity purified protein samples were treated with PNGase-F, which cleaves asparagine-linked oligosaccharides between the innermost N-acetylglucosamine and asparagine residues of glycoproteins [43]. N-glycosidase treatment eliminates the higher molecular weight band (14.5 kDa) present in the affinity purified AedaeGPA2 sample. This confirms that this subunit undergoes the predicted glycosylation during post-translation modification and that the lower molecular weight band (11.5 kDa) is the deglycosylated form of AedaeGPA2 (Figure 2B). The band migration in the affinity purified AedaeGPB5 sample was unaffected by treatment with N-glycosidase, which confirms the prediction that this subunit does not undergo N-linked glycosylation.

### Cloning of a Mosquito Glycoprotein Hormone Receptor cDNA and Sequence Analysis

**Figure 3:**
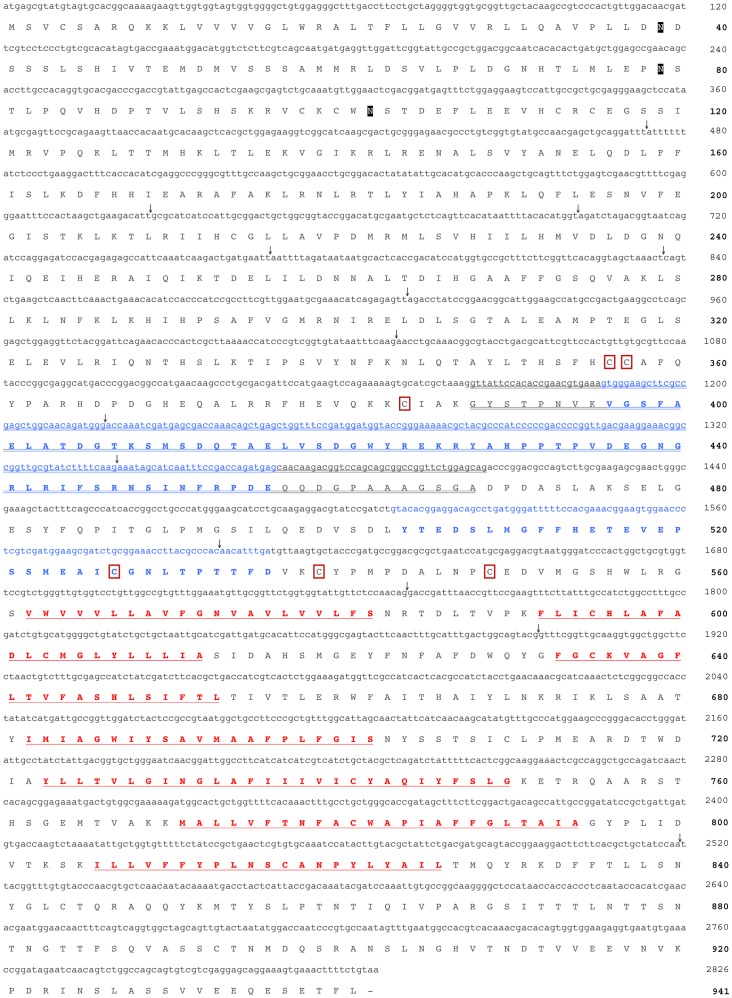
Cloned cDNA of *A. aegypti* glycoprotein hormone leucine-rich repeat containing receptor (AedaeLGR1) and deduced amino acid sequence (GenBank accession number: KF711859). Exon boundaries were determined by comparing the cloned cDNA against the *A. aegypti* genome and are denoted by downward pointing arrows. Novel nucleotide and corresponding amino acid sequence arising from unpredicted exons or incorrectly predicted introns in initial transcript prediction (GenBank accession EAT4421) are presented in bold blue font. Further, novel amino acid and nucleotide sequence differing from a second automated transcript prediction (GenBank accession XP_001649032) are indicated by a double underline. Predicted N-linked glycosylation sites are provided in white font outlined by a black box (Asn39, Asn79 and Asn102). Conserved cysteine residues located in the C-terminus of the ectodomain are highlighted by red outlined boxes (Cys_356_, Cys_357_, Cys_384_, Cys_527_, Cys_539_ and Cys_549_). Amino acid sequences forming the predicted seven hydrophobic membrane-spanning domains are denoted in bold red underlined font.

**Figure 4:**
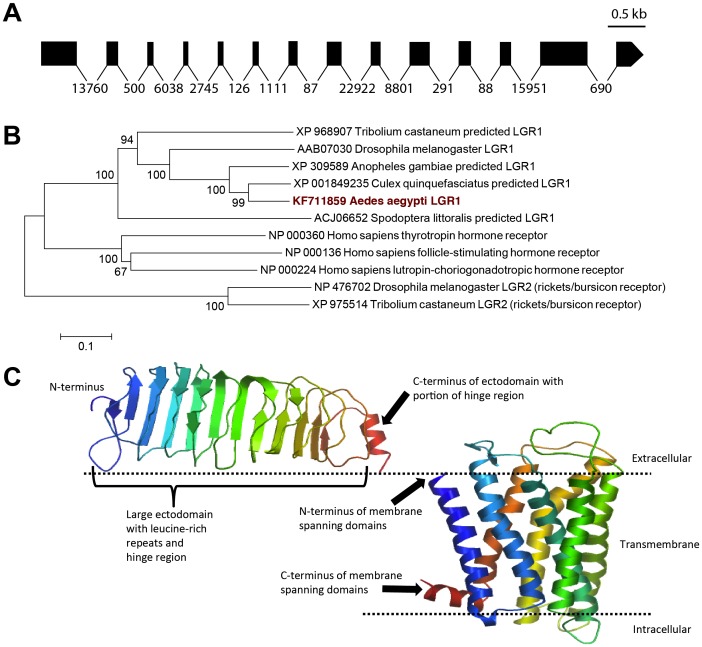
Gene model of open reading frame-spanning exons, phylogenetic analysis and structural modeling of the *Aedes aegypti* LGR1 receptor. In (A), legend bar denotes length of 0.5 kb and is proportional only for exons (shaded boxes). Introns (denoted by lines) have been standardized and are not proportional to size in order to optimize illustration of exon lengths. Numbers below lines adjoining exons denote predicted intron sizes based on *A. aegypti* genomic data. In (B), the evolutionary relationship was inferred using neighbor-joining method and statistical support was tested by 1000 bootstrap iterations where numbers adjacent to the branches denote the percentage of replicate trees in which the associated taxa clustered together. The tree is drawn to scale, with branch lengths indicative of the number of amino acid substitutions per site. In (C), a schematic representation of the major structural features of the AedaeLGR1, which includes the large ectodomain (containing leucine-rich repeats and hinge region) and seven transmembrane domains (see Results section). Note that portions of the protein lacking major predicted structural features are omitted from this schematic model. Given the low overall conservation within the hinge region of the ectodomain, the model of this feature is truncated and includes only the N-terminal portion of the hinge region.

Based on the predicted transcript and gene annotation of the leucine-rich repeat containing glycoprotein hormone receptor in *A. aegypti* (GenBank accession numbers XP 001649032 and EAT44221), we designed primers to amplify the entire predicted ORF (see Table 1). Following sequencing of multiple clones, it became clear that the cloned AedaeLGR1 cDNA differed from the previously predicted sequences (see Figure 3). These differences result in significantly larger ORF of 2826 bp that maps to 14 exons (Figure 4A), which span nearly 75 kb. This extended length is located over the protein region between the large ectodomain and hydrophobic transmembrane domains (see Figure S1). Interestingly, this extended region occurs, albeit with little or no sequence similarity, in other insect species (A. *aegypti*, *D. melanogaster* and *Tribolium castaneum*). Given the closer evolutionary relationship, the overall length of this poorly conserved region is similar in other mosquito species (*Culex quinquefasciatus* and *Anopheles gambiae*). In addition, we found several single base pair differences over the entire length of the cDNA, although these did not yield any amino acid differences based on the exon genome mapping predictions (data not shown). The AedaeLGR1 deduced protein sequence is comprised of 941 amino acids with an expected molecular weight of 104.94 kDa; however, given the existence of three putative N-linked glycosylation sites in the large ectodomain at residues Asn_39_, Asn_79_ and Asn_102_, the mature receptor protein likely has a greater molecular weight. Phylogenetic analysis confirms that the deduced protein sequence is the LGR1 homolog of the fruit fly GPA2/GPB5 receptor, DLGR1 [6], [12], with predicted sequences in other mosquito species as well as in the bettle, *Tribolium castaneum*. In addition, the *A. aegypti* LGR1 has high sequence similarity with the mammalian receptor proteins including gonadotropin and thyrotropin receptors and the next closest insect receptor protein being the bursicon receptor (Figure 4B). Using the Swiss Model server, we modeled the three dimensional structure of the large ectodomain that comprises approximately 60% of the receptor protein and includes ten leucine-rich repeats. We also modeled the region of the protein including the seven transmembrane-spanning hydrophobic domains. We assembled a schematic of these major structural features based on the predicted three-dimensional models of these two characteristic regions of this class of glycoprotein hormone receptor (Figure 4C).

### Stage Specific Expression Analysis of the Glycoprotein Hormone Subunits

**Figure 5:**
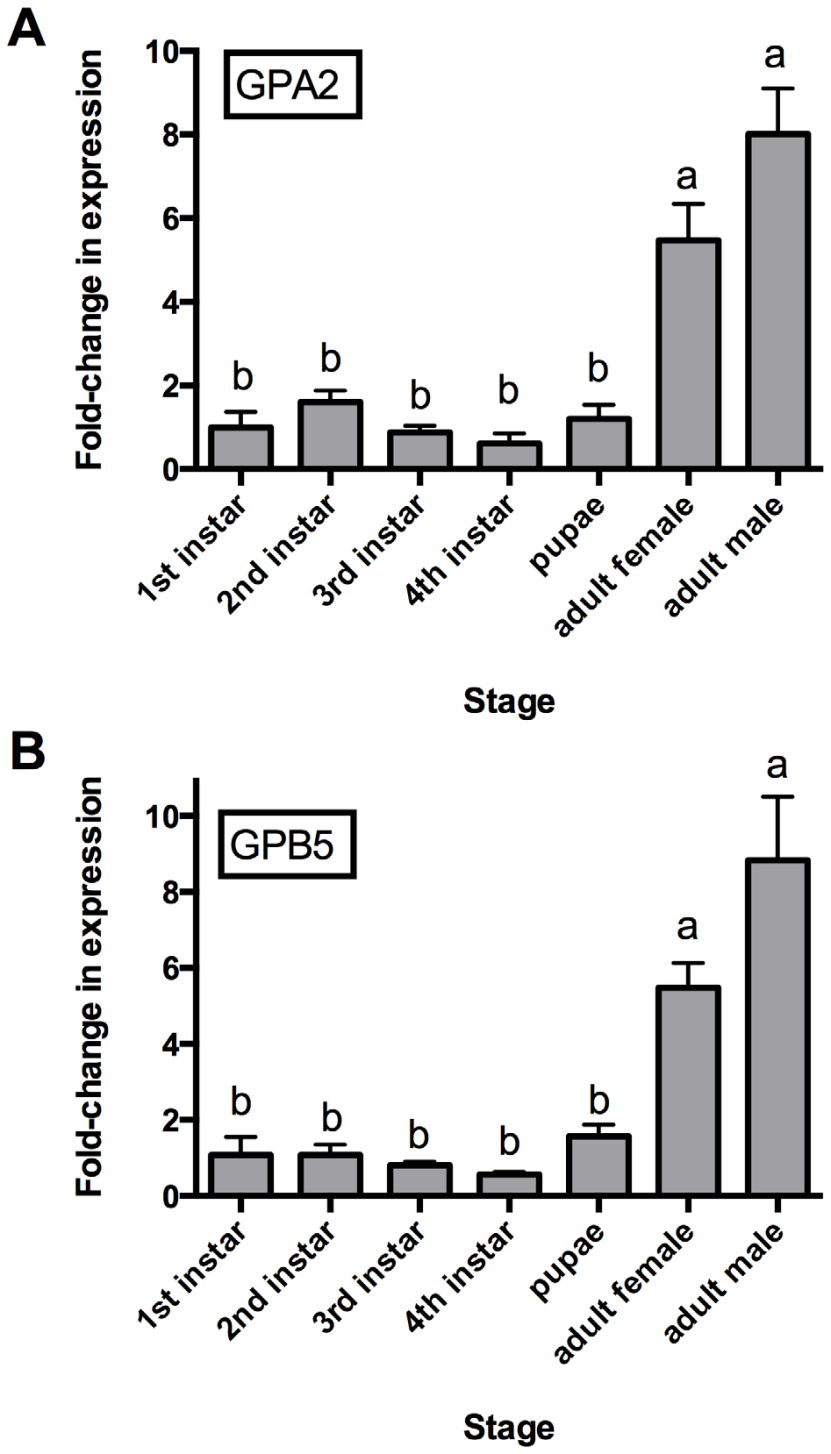
Developmental transcript expression profile of glycoprotein hormone subunits. Expression analysis of glycoprotein hormone (A) GPA2 and (B) GPB5 subunit transcripts by qRT-PCR. Both subunit transcripts have significantly higher abundance during the adult developmental stage. Moreover, adult males show marginally higher levels of each transcript relative to adult females although this difference was not significant. Expression of each subunit was graphed relative to expression levels in 1^st^ instar larvae.

To infer possible physiological roles for the mosquito glycoprotein hormone AedaeGPA2/GPB5, we examined the transcript expression profile of the two subunits forming the mature hormone during each post-embryonic developmental stage. We detected expression of both subunit transcripts in all developmental stages examined; however, both AedaeGPA2 (Figure 5A) and AedaeGPB5 (Figure 5B) transcripts were significantly up regulated (by five to eight-fold) in both male and female adult stage mosquitoes relative to the pupal and larval stages.

### Temporal and Adult Spatial Expression Analysis of AedaeLGR1

**Figure 6:**
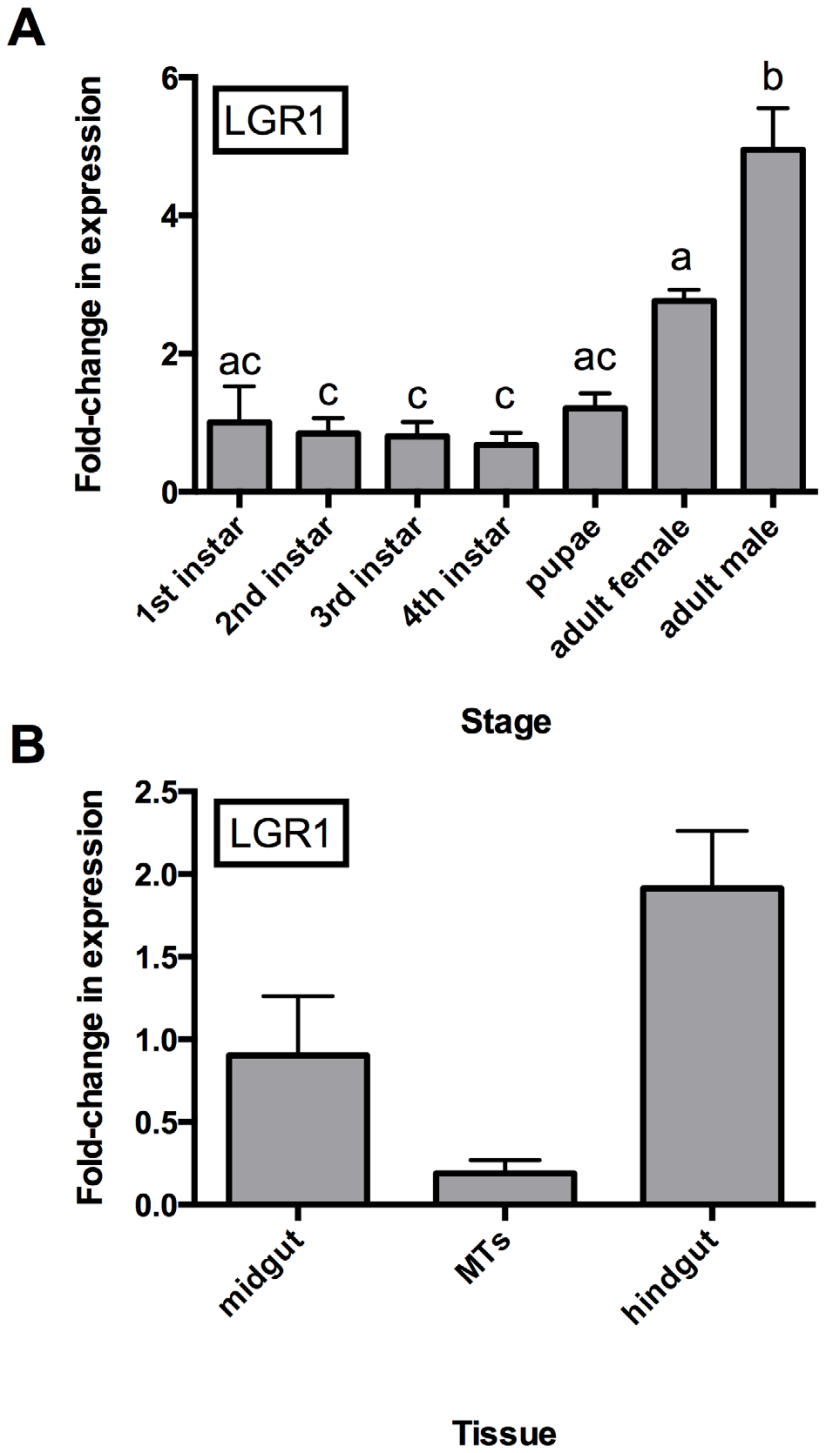
Developmental and spatial transcript expression profile of the GPA2/GPB5 glycoprotein hormone receptor (LGR1) gene determined by qRT-PCR. In (A), AedaeLGR1 transcript levels were examined over developmental stages with significantly higher abundance occurring in male adults relative to adult female, pupal and larval stages. Expression of AedaeLGR1 in adult females is also significantly elevated relative to 2^nd^ to 4^th^ instar larvae. In (B), expression was examined in selected alimentary canal tissues in adult stage insects. The AedaeLGR1 transcript expression is significantly higher in hindgut relative to Malpighian tubules. Expression was graphed relative to whole body mixed sex adult expression levels.

In order to provide further evidence for inferring possible physiological activities governed by this glycoprotein hormone signaling system in the mosquito, we examined expression in all post-embryonic stages and tissue-specific expression in selected adult alimentary canal tissues for the prospective *A. aegypti* GPA2/GPB5 receptor, AedaeLGR1. Expression of AedaeLGR1 was detected in all post-embryonic stages examined, with expression in adult males being significantly higher than adult female, pupal and all larval stage expression levels (Figure 6A). This expression level was approximately five-fold higher than levels found in pupal and larval stages and nearly two-fold higher than adult female levels. Interestingly, although not as dramatic, adult female expression was also elevated, and was significantly higher than expression in second, third and fourth larval stages (Figure 6A).

Selected tissue-specific expression was examined in adult stage *A. aegypti*. With an interest to examine a possible involvement of this neuroendocrine system related to ionic and osmotic balance in the mosquito, we examined some epithelial tissues, including those that comprise the insect excretory system. Relative to whole body expression levels in adults, expression was not significantly different in any particular tissue, but trends signify that expression was lower in the Malpighian tubules and elevated in the hindgut tissue (Figure 6B). Notably, however, the transcript abundance of AedaeLGR1 in the hindgut was significantly higher than levels detected in the Malpighian tubules.

### SIET Analysis of Hindgut Following Treatment with GPA2/GPB5

**Figure 7:**
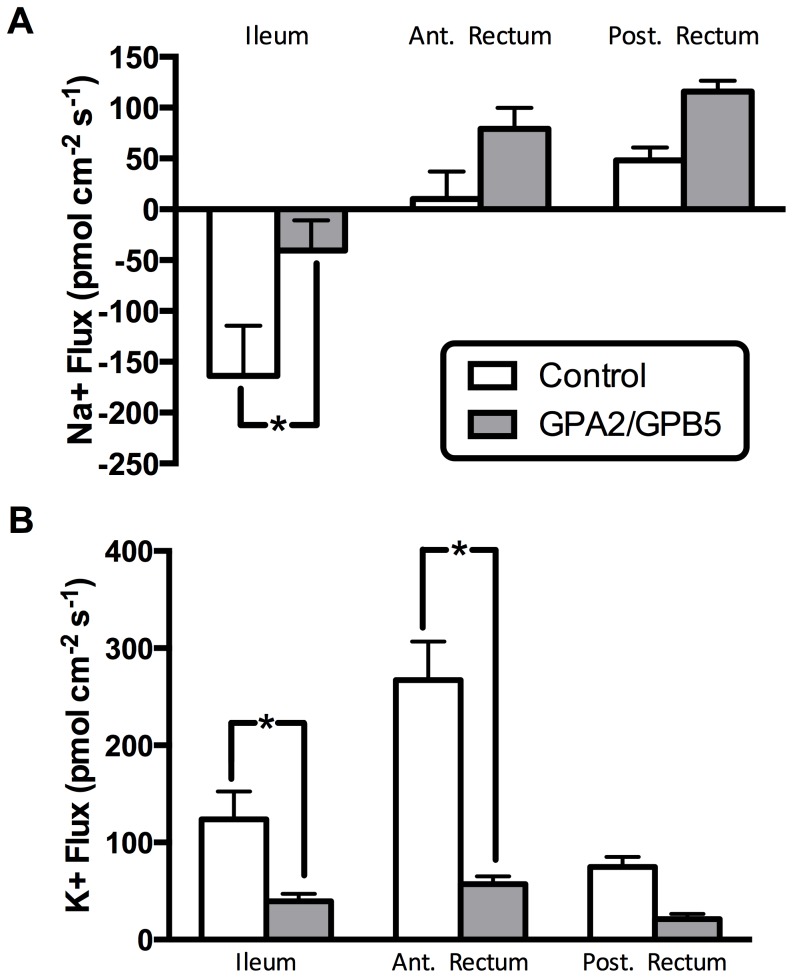
Cation transport across the hindgut of adult *Aedes aegypti* is modulated by the recombinant glycoprotein hormone, GPA2/GPB5. In (A), lumen directed transport (i.e. secretion) of Na^+^ in the ileum is significantly inhibited by AedaeGPA2/GPB5. In contrast, no significant change in Na^+^ flux was observed in the anterior or posterior rectum. Conversely in (B), haemolymph directed K^+^ transport (i.e. absorption) is significantly inhibited by AedaeGPA2/GPB5 in the ileum and anterior portion of the rectum.

Exposure to recombinant Aedae GPA2/GPB5 (200 nM) had a significant effect on Na^+^ transport only in the ileum, where it reduced Na^+^ secretion 75% below the control level (Figure 7A). The slight increases in Na^+^ absorption in both halves of the rectum upon glycoprotein hormone exposure were not statistically significant (Figure 7A). AedaeGPA2/GPB5 (200 nM) exposure also caused significant reductions in K^+^ absorption across the ileum and anterior rectum epithelia (Figure 7B). K^+^ absorption was significantly altered by treatment with recombinant Aedae GPA2/GPB5 where it decreased by 68% in the ileum and by 79% in the anterior rectum relative to the control level. A non-significant but similar trending reduction in K^+^ absorption was measured across the posterior rectum epithelia of preparations exposed to the recombinant Aedae GPA2/GPB5 (Figure 7B).

## Discussion

The relatively recent molecular identification of the evolutionarily conserved GPA2/GPB5 glycoprotein hormone signaling system that evolved prior to the emergence of bilateral metazoan organisms [12], [44] warrants further research into elucidating physiological actions regulated by this heterodimeric glycoprotein hormone in insects. In the fruit fly *D. melanogaster*, the components of this signaling system have been well characterized. This includes the identification and functional validation of the leucine-rich repeat-containing GPCR, *D. melanogaster* LGR1 [6], [12], [45]. With structural similarities to the limited cloned receptor homologs in insects, found exclusively in *D. melanogaster* [4], [5], [12], [45], [46], and human homologs including the TSH, LH/hCG and FSH receptors [47]–[50], the mosquito LGR1 has a large ectodomain that comprises the majority (∼60%) of the entire protein and contains ten leucine-rich repeats (LRRs). The LRRs are conserved with those previously identified in the *D. melanogaster* (see Figure S1) [12]. Interestingly, our structural modeling of the large ectodomain of AedaeLGR1 shows similarities to the recently resolved crystal structure of the human FSH receptor ectodomain [9], where the enigmatic hinge region forms an integral component of the ectodomain rather than a distinct structural entity as previously believed [51]. Moreover, the hinge region of AedaeLGR1 contains six cysteine residues flanking the LRR domain on the C-terminus (Cys_356_, Cys_357_, Cys_384_, Cys_527_, Cys_539_ and Cys_549_; see Figure 3 and Figure S1) and four cysteine residues flank the LRR domain on the N-terminus (Cys_98_, Cys_100_, Cys_113_ and Cys_115_; see Figure S1) and are similarly positioned to those found in related receptors [9], [12], [52], which likely reflects their role in disulfide bond formation. Finally, AedaeLGR1 contains classical features that typify members of the large superfamily of GPCRs, which includes seven transmembrane domains, and additionally, three predicted N-linked glycosylation sites (Asn_39_, Asn_79_ and Asn_102_; see Figure 3 and Figure S1), features that have been demonstrated previously in closely related receptor proteins in insects [12], [45].

The glycoprotein hormone subunits have also been identified in insects, including *D. melanogaster* GPA2 [6] and GPB5 [2] and gene predictions from several other insects with genome database availability. The mosquito glycoprotein hormone subunits each contain ten conserved cysteine residues of critical importance for disulfide bridge formation and important for cystine-knot formation. The AedaeGPA2 and AedaeGPB5 subunits share ten conserved cysteine residues with the classic human GPA1 subunit and both human and fly GPA2 and GPB5 subunits [2], [6]. This is in contrast to the classic GPB1-4 subunits, which are comprised of 12 cysteine residues that form an additional disulfide bridge yielding the seat belt structure [2]. Interestingly, while both human GPA2 and GPB5 subunits undergo N-linked glycosylation [2], we show that only *A. aegypti* GPA2 undergoes glycosylation, whereas in contrast, no predicted or experimentally determined glycosylation was evident with AedaeGPB5. This observation is consistent with the subunit counterparts in *D. melanogaster* with GPA2 undergoing glycosylation whereas this post-translation modification does not occur with the fly GPB5 subunit [6].

The fruit fly LGR1 transcript has been shown to be six times more abundant in adult males compared to adult females, and it is also expressed early in development, with expression detected as early as 8–16 hours after oviposition [12]. This led to the suggestion that this receptor protein may be dually involved in developmental and reproductive biology, especially spermatogenesis or other male-specific processes related to reproduction [12]. However, examination of FlyAtlas expression data for the fruit fly LGR1 transcript reveals greatest enrichment within the adult salivary glands and hindgut, but not in any tissues of the male or female reproductive systems [15]. In partial agreement with these expression profiles in *D. melanogaster*, we identified that expression of the putative *A. aegypti* LGR1 is also increased in the adult stages, with greatest enrichment in males. Within the adult stage mosquito, we examined AedaeLGR1 expression in selected tissues of the alimentary canal and compared expression relative to the whole adult expression profile to determine tissue-specific enrichment. We identified increased expression in the hindgut, which was significantly higher than expression levels in the Malpighian tubules and this result is similar to the enrichment profile in adult fruit fly hindgut relative to the tubules [15]. The transcripts of the glycoprotein hormone subunits are also significantly elevated during the adult stage in both males and females, relative to pupal and larval stages. In *D. melanogaster*, cells most strongly expressing both of the fly glycoprotein hormone subunits are relatively large neurons having characteristics of neurosecretory cells that are localized within the first four abdominal neuromeres in larval and adult stages [7]. Taken together, these results suggest that the GPA2/GPB5 hormonal system may participate in the control of ion and water balance by regulating the transport properties of epithelial tissues in the insect alimentary canal.

With a prospective role in controlling transporting epithelial tissues in the mosquito gut, we utilized SIET to examine if the purified recombinant mosquito glycoprotein hormone could modulate transport of the principal cations, Na^+^ and K^+^. In this study, we focused on examining physiological activity on the adult hindgut, since this was the most practical starting point considering the receptor transcript enrichment observed in adult hindgut tissue. Based on preliminary scans along the entire length of the hindgut, we separated our measurements into bins reflecting three segments of the hindgut (ileum, anterior rectum and posterior rectum). In controls, Na^+^ was normally secreted by the ileum, while Na^+^ transport was slightly directed towards the haemolymph, and thus absorbed, by the rectum. Interestingly, treatment by the recombinant mosquito glycoprotein hormone led to a decrease in net secretion of Na^+^ by the ileum as is evident by a significant decrease in lumen-directed Na^+^ flux and caused a mild increase in absorption by both the anterior and posterior regions of the hindgut. Examination of the other principal cation in control treatments, K^+^ was normally absorbed across all regions of the hindgut, with greatest K^+^ flux occurring in the anterior rectum followed by the ileum. Interestingly, treatment with the recombinant mosquito glycoprotein hormone decreased K^+^ absorption in all hindgut regions examined, with significantly reduced K^+^ flux in the ileum and anterior rectum. Taken together, these results suggest that the physiological role of the glycoprotein hormone *in vivo* may be to inhibit natriuresis and promotes kaliuresis. This may be an important physiological adaptation for recently emerged adults of both sexes as they clear excess cations such as K^+^ derived from bacteria, algae and other small organisms ingested during larval development. Later, this control mechanism could also play an important function to ensure that the blood-gorged female can successfully remove excess K^+^ and conserve the limited Na^+^ as blood cells are lysed and digested. Blood-feeding insects such as mosquitoes must effectively switch their regulatory strategies for ion balance between the short and long-term time periods, which precede and follow blood meal engorgement. Immediately after feeding on vertebrate blood (or as the insect is still taking its fill), excess ions and water that must be removed from the blood are mainly made up of Na^+^ arising from the plasma portion of the blood meal, which is known as the peak period of diuresis in mosquitoes [53]. However, as the blood cells are continually lysed and assimilated, the level of excess K^+^ increases and Na^+^ decreases, leading to the elimination of K^+^-rich, Na^+^-poor urine [53]. The production of this later form of urine requires the action of a hormone working similarly to the mosquito heterodimeric glycoprotein hormone revealed in this study. Based on the *in vitro* physiological activities elucidated, AedaeGPA2/GPB5 has a potential *in vivo* role to decrease excretion of Na^+^ while simultaneosly increasing excretion of K^+^ ions. Considering the *A. aegypti* adult hindgut tissue distribution and subcellular localization of the apically situated V-type H^+^-ATPase and basolaterally situated P-type Na^+^/K^+^ ATPase reported previously [54], suggests the mosquito GPA2/GPB5 may mediate its effects by regulating activity of these two transporters to enable recycling of Na^+^ ions at the haemolymph surface and eliminating excess K^+^. In comparison to the model developed for the locust hindgut [55], GPA2/GPB5 inhibits K^+^ absorption and thus has effects opposite to chloride transport stimulating hormone (CTSH), which stimulates K^+^ absorption. In further support of this difference, CTSH activity arises from extracts purified from the locust corpus cardiacum [56], a region where no GPA2 or GPB5 immunoreactivity has been localized in the fruit fly, *D. melanogaster* [7]. It is anticipated that further research will help better understand the physiological roles and identify additional tissue targets for this evolutionary old neurohormone signaling system in insects.

## Supporting Information

Figure S1 Multiple sequence alignment of predicted or empirically determined insect LGR1 receptors. Major features typifying this class of receptor protein are demarcated on the *Aedes aegypti* sequence. These features include the leucine-rich repeats (LRRs) denoted by orange boxes and labeled L1−L10; the three predicted glycosylation sites denoted by purple boxes; the four N-terminal and six C-terminal cysteine residues which flank the LRRs and likely form disulfide bridges are denoted by green boxes; the hinge region between the LRRs and the transmembrane domains is denoted by a blue box; the transmembrane domains are denoted by red boxes and the cytoplasmic and extracellular regions of the transmembrane region are denoted by pink boxes and named accordingly.
(TIF)

## References

[1] Pierce JG, Parsons TF (1981) Glycoprotein Hormones - Structure and Function. Annual Review of Biochemistry 50: 465–495. 10.1146/annurev.bi.50.070181.002341 6267989

[2] Hsu SY, Nakabayashi K, Bhalla A (2002) Evolution of glycoprotein hormone subunit genes in bilateral metazoa: identification of two novel human glycoprotein hormone subunit family genes, GPA2 and GPB5. Mol Endocrinol 16: 1538–1551. 12089349 10.1210/mend.16.7.0871

[3] Nakabayashi K, Matsumi H, Bhalla A, Bae J, Mosselman S, et al (2002) Thyrostimulin, a heterodimer of two new human glycoprotein hormone subunits, activates the thyroid-stimulating hormone receptor. J Clin Invest 109: 1445–1452. 12045258 10.1172/JCI14340 PMC150994

[4] Mendive FM, Van Loy T, Claeysen S, Poels J, Williamson M, et al (2005) Drosophila molting neurohormone bursicon is a heterodimer and the natural agonist of the orphan receptor DLGR2. FEBS Lett 579: 2171–2176. 15811337 10.1016/j.febslet.2005.03.006

[5] Luo CW, Dewey EM, Sudo S, Ewer J, Hsu SY, et al (2005) Bursicon, the insect cuticle-hardening hormone, is a heterodimeric cystine knot protein that activates G protein-coupled receptor LGR2. Proc Natl Acad Sci U S A 102: 2820–2825. 15703293 10.1073/pnas.0409916102 PMC549504

[6] Sudo S, Kuwabara Y, Park JI, Hsu SY, Hsueh AJ (2005) Heterodimeric fly glycoprotein hormone-alpha2 (GPA2) and glycoprotein hormone-beta5 (GPB5) activate fly leucine-rich repeat-containing G protein-coupled receptor-1 (DLGR1) and stimulation of human thyrotropin receptors by chimeric fly GPA2 and human GPB5. Endocrinology 146: 3596–3604. 15890769 10.1210/en.2005-0317

[7] Sellami A, Agricola HJ, Veenstra JA (2011) Neuroendocrine cells in Drosophila melanogaster producing GPA2/GPB5, a hormone with homology to LH, FSH and TSH. Gen Comp Endocrinol 170: 582–588. 21118692 10.1016/j.ygcen.2010.11.015

[8] Nakabayashi K, Kudo M, Hsueh AJ, Maruo T (2003) Activation of the luteinizing hormone receptor in the extracellular domain. Mol Cell Endocrinol 202: 139–144. 12770743 10.1016/s0303-7207(03)00075-3

[9] Jiang X, Liu H, Chen X, Chen PH, Fischer D, et al (2012) Structure of follicle-stimulating hormone in complex with the entire ectodomain of its receptor. Proc Natl Acad Sci U S A 109: 12491–12496. 22802634 10.1073/pnas.1206643109 PMC3411987

[10] Royer J, Lefevre-Minisini A, Caltabiano G, Lacombe T, Malthiery Y, et al (2008) The cloned equine thyrotropin receptor is hypersensitive to human chorionic gonadotropin; identification of three residues in the extracellular domain involved in ligand specificity. Endocrinology 149: 5088–5096. 18535103 10.1210/en.2008-0423

[11] Fan QR, Hendrickson WA (2007) Assembly and structural characterization of an authentic complex between human follicle stimulating hormone and a hormone-binding ectodomain of its receptor. Mol Cell Endocrinol 260–262: 73–82. 10.1016/j.mce.2005.12.055 PMC2012943 17045735

[12] Hauser F, Nothacker HP, Grimmelikhuijzen CJ (1997) Molecular cloning, genomic organization, and developmental regulation of a novel receptor from Drosophila melanogaster structurally related to members of the thyroid-stimulating hormone, follicle-stimulating hormone, luteinizing hormone/choriogonadotropin receptor family from mammals. J Biol Chem 272: 1002–1010. 8995395 10.1074/jbc.272.2.1002

[13] Fraenkel G, Hsiao C (1962) Hormonal and nervous control of tanning in the fly. Science 138: 27–29. 13894424 10.1126/science.138.3536.27

[14] Fraenkel G, Hsiao C (1963) Tanning in the Adult Fly: A New Function of Neurosecretion in the Brain. Science 141: 1057–1058. 14043350 10.1126/science.141.3585.1057

[15] Chintapalli VR, Wang J, Dow JA (2007) Using FlyAtlas to identify better Drosophila melanogaster models of human disease. Nat Genet 39: 715–720. 17534367 10.1038/ng2049

[16] Oishi A, Gengyo-Ando K, Mitani S, Mohri-Shiomi A, Kimura KD, et al (2009) FLR-2, the glycoprotein hormone alpha subunit, is involved in the neural control of intestinal functions in Caenorhabditis elegans. Genes Cells 14: 1141–1154. 19735483 10.1111/j.1365-2443.2009.01341.x

[17] Petersen TN, Brunak S, von Heijne G, Nielsen H (2011) SignalP 4.0: discriminating signal peptides from transmembrane regions. Nat Methods 8: 785–786. 21959131 10.1038/nmeth.1701

[18] Blom N, Sicheritz-Ponten T, Gupta R, Gammeltoft S, Brunak S (2004) Prediction of post-translational glycosylation and phosphorylation of proteins from the amino acid sequence. Proteomics 4: 1633–1649. 15174133 10.1002/pmic.200300771

[19] Wistrand M, Kall L, Sonnhammer EL (2006) A general model of G protein-coupled receptor sequences and its application to detect remote homologs. Protein Sci 15: 509–521. 16452613 10.1110/ps.051745906 PMC2249772

[20] Tamura K, Peterson D, Peterson N, Stecher G, Nei M, et al (2011) MEGA5: molecular evolutionary genetics analysis using maximum likelihood, evolutionary distance, and maximum parsimony methods. Mol Biol Evol 28: 2731–2739. 21546353 10.1093/molbev/msr121 PMC3203626

[21] Saitou N, Nei M (1987) The neighbor-joining method: a new method for reconstructing phylogenetic trees. Mol Biol Evol 4: 406–425. 3447015 10.1093/oxfordjournals.molbev.a040454

[22] Jones DT, Taylor WR, Thornton JM (1992) The rapid generation of mutation data matrices from protein sequences. Comput Appl Biosci 8: 275–282. 1633570 10.1093/bioinformatics/8.3.275

[23] Felsenstein J (1985) Confidence-Limits on Phylogenies - an Approach Using the Bootstrap. Evolution 39: 783–791. 28561359 10.1111/j.1558-5646.1985.tb00420.x

[24] Arnold K, Bordoli L, Kopp J, Schwede T (2006) The SWISS-MODEL workspace: a web-based environment for protein structure homology modelling. Bioinformatics 22: 195–201. 16301204 10.1093/bioinformatics/bti770

[25] Guex N, Peitsch MC (1997) SWISS-MODEL and the Swiss-PdbViewer: an environment for comparative protein modeling. Electrophoresis 18: 2714–2723. 9504803 10.1002/elps.1150181505

[26] Schwede T, Kopp J, Guex N, Peitsch MC (2003) SWISS-MODEL: An automated protein homology-modeling server. Nucleic Acids Res 31: 3381–3385. 12824332 10.1093/nar/gkg520 PMC168927

[27] Shimamura T, Hiraki K, Takahashi N, Hori T, Ago H, et al (2008) Crystal structure of squid rhodopsin with intracellularly extended cytoplasmic region. Journal of Biological Chemistry 283: 17753–17756. 18463093 10.1074/jbc.C800040200 PMC2440622

[28] Paluzzi JP, Park Y, Nachman RJ, Orchard I (2010) Isolation, expression analysis, and functional characterization of the first antidiuretic hormone receptor in insects. Proc Natl Acad Sci U S A 107: 10290–10295. 20479227 10.1073/pnas.1003666107 PMC2890494

[29] Paluzzi JP, Young P, Defferrari MS, Orchard I, Carlini CR, et al (2012) Investigation of the potential involvement of eicosanoid metabolites in anti-diuretic hormone signaling in Rhodnius prolixus. Peptides 34: 127–134. 22079222 10.1016/j.peptides.2011.10.025

[30] Roucou X, Giannopoulos PN, Zhang Y, Jodoin J, Goodyer CG, et al (2005) Cellular prion protein inhibits proapoptotic Bax conformational change in human neurons and in breast carcinoma MCF-7 cells. Cell Death Differ 12: 783–795. 15846375 10.1038/sj.cdd.4401629

[31] Paluzzi JP, O'Donnell M J (2012) Identification, spatial expression analysis and functional characterization of a pyrokinin-1 receptor in the Chagas’ disease vector, Rhodnius prolixus. Molecular and Cellular Endocrinology 363: 36–45. 22820129 10.1016/j.mce.2012.07.007

[32] Bradford MM (1976) A rapid and sensitive method for the quantitation of microgram quantities of protein utilizing the principle of protein-dye binding. Anal Biochem 72: 248–254. 942051 10.1016/0003-2697(76)90527-3

[33] Pfaffl MW, Tichopad A, Prgomet C, Neuvians TP (2004) Determination of stable housekeeping genes, differentially regulated target genes and sample integrity: BestKeeper–Excel-based tool using pair-wise correlations. Biotechnol Lett 26: 509–515. 15127793 10.1023/b:bile.0000019559.84305.47

[34] Andersen CL, Jensen JL, Orntoft TF (2004) Normalization of real-time quantitative reverse transcription-PCR data: a model-based variance estimation approach to identify genes suited for normalization, applied to bladder and colon cancer data sets. Cancer Res 64: 5245–5250. 15289330 10.1158/0008-5472.CAN-04-0496

[35] Vandesompele J, De Preter K, Pattyn F, Poppe B, Van Roy N, et al (2002) Accurate normalization of real-time quantitative RT-PCR data by geometric averaging of multiple internal control genes. Genome Biol 3: RESEARCH0034. 12184808 10.1186/gb-2002-3-7-research0034 PMC126239

[36] Thomas RC (1978) Ion-Sensitive Intracellular Microelectrodes. How to Make and Use Them. London: Academic Press.

[37] Ammann D, Chao PS, Simon W (1987) Valinomycin-based K+ selective microelectrodes with low electrical membrane resistance. Neurosci Lett 74: 221–226. 3574760 10.1016/0304-3940(87)90153-4

[38] Messerli MA, Kurtz I, Smith PJ (2008) Characterization of optimized Na+ and Cl- liquid membranes for use with extracellular, self-referencing microelectrodes. Analytical and Bioanalytical Chemistry 390: 1355–1359. 18193410 10.1007/s00216-007-1804-z

[39] Jayakannan M, Babourina O, Rengel Z (2011) Improved measurements of Na+ fluxes in plants using calixarene-based microelectrodes. Journal of Plant Physiology 168: 1045–1051. 21256620 10.1016/j.jplph.2010.12.006

[40] Naikkhwah W, O'Donnell MJ (2012) Phenotypic plasticity in response to dietary salt stress: Na+ and K+ transport by the gut of Drosophila melanogaster larvae. J Exp Biol 215: 461–470. 22246255 10.1242/jeb.064048

[41] Lide DR (2002) Handbook of Chemistry and Physics. Boca Raton, FL: CRC Press.

[42] Vitt UA, Hsu SY, Hsueh AJ (2001) Evolution and classification of cystine knot-containing hormones and related extracellular signaling molecules. Mol Endocrinol 15: 681–694. 11328851 10.1210/mend.15.5.0639

[43] Plummer TH Jr, Elder JH, Alexander S, Phelan AW, Tarentino AL (1984) Demonstration of peptide:N-glycosidase F activity in endo-beta-N-acetylglucosaminidase F preparations. J Biol Chem 259: 10700–10704. 6206060

[44] Vibede N, Hauser F, Williamson M, Grimmelikhuijzen CJ (1998) Genomic organization of a receptor from sea anemones, structurally and evolutionarily related to glycoprotein hormone receptors from mammals. Biochem Biophys Res Commun 252: 497–501. 9826559 10.1006/bbrc.1998.9661

[45] Nishi S, Hsu SY, Zell K, Hsueh AJ (2000) Characterization of two fly LGR (leucine-rich repeat-containing, G protein-coupled receptor) proteins homologous to vertebrate glycoprotein hormone receptors: constitutive activation of wild-type fly LGR1 but not LGR2 in transfected mammalian cells. Endocrinology 141: 4081–4090. 11089539 10.1210/endo.141.11.7744

[46] Eriksen KK, Hauser F, Schiott M, Pedersen KM, Sondergaard L, et al (2000) Molecular cloning, genomic organization, developmental regulation, and a knock-out mutant of a novel leu-rich repeats-containing G protein-coupled receptor (DLGR-2) from Drosophila melanogaster. Genome Res 10: 924–938. 10899142 10.1101/gr.10.7.924 PMC310913

[47] Nagayama Y, Kaufman KD, Seto P, Rapoport B (1989) Molecular cloning, sequence and functional expression of the cDNA for the human thyrotropin receptor. Biochem Biophys Res Commun 165: 1184–1190. 2558651 10.1016/0006-291x(89)92727-7

[48] Misrahi M, Loosfelt H, Atger M, Sar S, Guiochon-Mantel A, et al (1990) Cloning, sequencing and expression of human TSH receptor. Biochem Biophys Res Commun 166: 394–403. 2302212 10.1016/0006-291x(90)91958-u

[49] Jia XC, Oikawa M, Bo M, Tanaka T, Ny T, et al (1991) Expression of human luteinizing hormone (LH) receptor: interaction with LH and chorionic gonadotropin from human but not equine, rat, and ovine species. Mol Endocrinol 5: 759–768. 1922095 10.1210/mend-5-6-759

[50] Tilly JL, Aihara T, Nishimori K, Jia XC, Billig H, et al (1992) Expression of recombinant human follicle-stimulating hormone receptor: species-specific ligand binding, signal transduction, and identification of multiple ovarian messenger ribonucleic acid transcripts. Endocrinology 131: 799–806. 1322283 10.1210/endo.131.2.1322283

[51] Mueller S, Jaeschke H, Gunther R, Paschke R (2010) The hinge region: an important receptor component for GPHR function. Trends Endocrinol Metab 21: 111–122. 19819720 10.1016/j.tem.2009.09.001

[52] Bruysters M, Verhoef-Post M, Themmen AP (2008) Asp330 and Tyr331 in the C-terminal cysteine-rich region of the luteinizing hormone receptor are key residues in hormone-induced receptor activation. J Biol Chem 283: 25821–25828. 18641392 10.1074/jbc.M804395200 PMC2533784

[53] Williams JC, Hagedorn HH, Beyenbach KW (1983) Dynamic Changes in Flow-Rate and Composition of Urine during the Post-Bloodmeal Diuresis in Aedes-Aegypti (L). Journal of Comparative Physiology 153: 257–265.

[54] Patrick ML, Aimanova K, Sanders HR, Gill SS (2006) P-type Na+/K+-ATPase and V-type H+-ATPase expression patterns in the osmoregulatory organs of larval and adult mosquito Aedes aegypti. J Exp Biol 209: 4638–4651. 17114398 10.1242/jeb.02551

[55] Phillips JE, Audsley N (1995) Neuropeptide control of ion and fluid transport across locust hindgut. American Zoologist 35: 503–514.

[56] Spring JH, Phillips JE (1980) Studies on locust rectum. II. Identification of specific ion-transport processes regulated by corpora cardiaca and cyclic AMP. J Exp Biol 86: 225–236.

