# Supplementary figures and images for: The Heterodimeric Glycoprotein Hormone, GPA2/GPB5, Regulates Ion Transport across the Hindgut of the Adult Mosquito, Aedes aegypti
Source: PLoS One. 2014 Jan 20;9(1):e86386. doi: 10.1371/journal.pone.0086386 (PMC3896475; doi:10.1371/journal.pone.0086386)

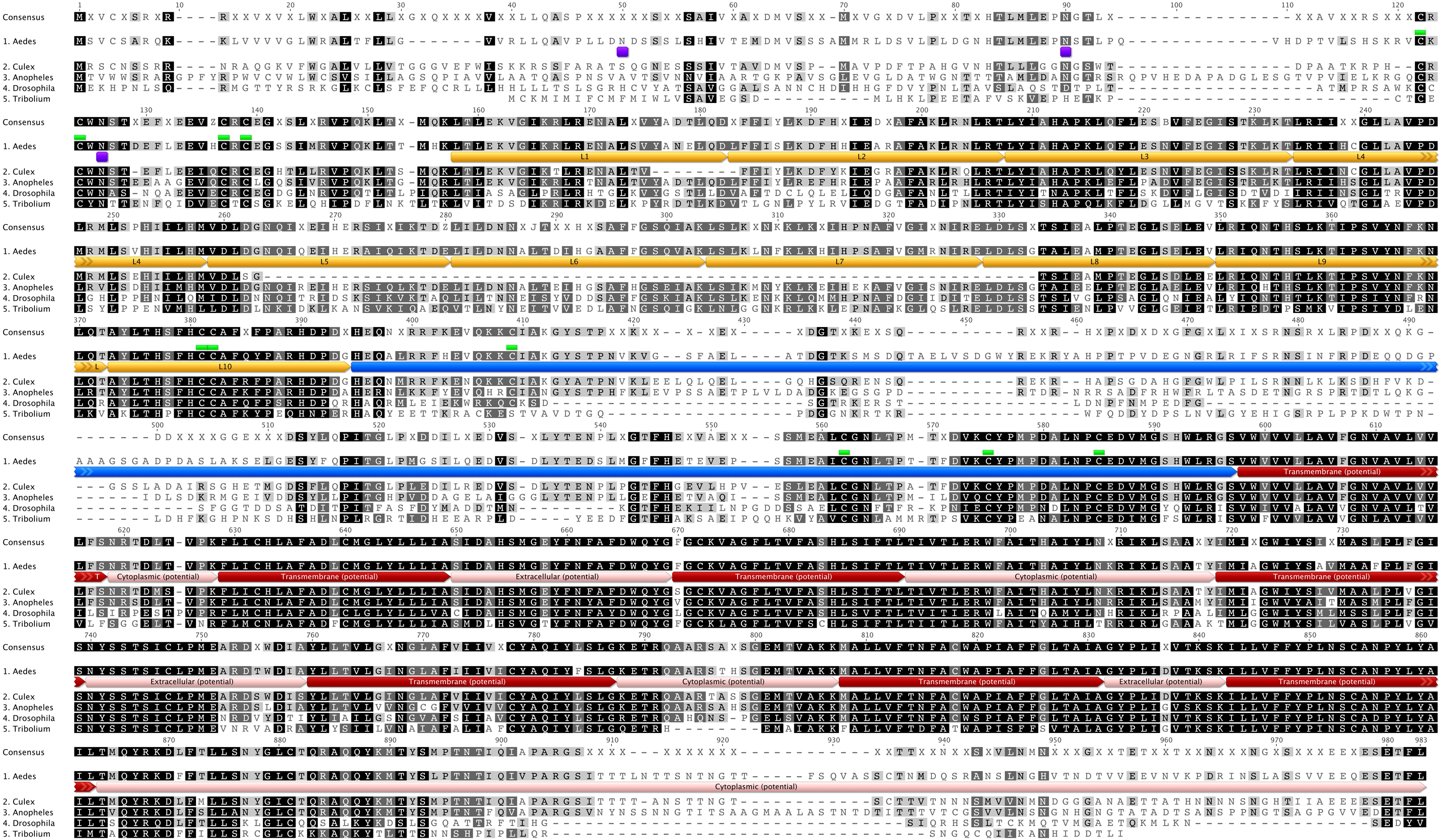

Supplement: Figure S1 — Multiple sequence alignment of predicted or empirically determined insect LGR1 receptors. Major features typifying this class of receptor protein are demarcated on the Aedes aegypti sequence. These features include the leucine-rich repeats (LRRs) denoted by orange boxes and labeled L1−L10; the three predicted glycosylation sites denoted by purple boxes; the four N-terminal and six C-terminal cysteine residues which flank the LRRs and likely form disulfide bridges are denoted by green boxes; the hinge region between the LRRs and the transmembrane domains is denoted by a blue box; the transmembrane domains are denoted by red boxes and the cytoplasmic and extracellular regions of the transmembrane region are denoted by pink boxes and named accordingly. (TIF) [file pone.0086386.s001.tif]
